# Exploring the aesthetic cognition and artistic acceptance of AIGC-generated urban sculptures: A structural equation modeling and visual content analysis approach

**DOI:** 10.1371/journal.pone.0344501

**Published:** 2026-03-12

**Authors:** Hao Fang, Bowen Li, Ziwen Zhou, Mu Li, Huasheng Lai, Yihao Zheng, Bin Hu, Weichang Chen

**Affiliations:** 1 Institute of Fine Arts and Design, Quanzhou Normal University, Quanzhou, China; 2 School of Fine Arts, Jiangxi Science & Technology Normal University, Nanchang, China; 3 Product Design Department, School of the Arts, Universiti Sains Malaysia, Penang, Malaysia; 4 Fuzhou University Xiamen College of Art and Design, Xiamen, China; 5 School of Design and Art, Southwest Forestry University, Kunming, China; Federal University of Paraiba, BRAZIL

## Abstract

As artificial intelligence–generated content (AIGC) becomes increasingly integrated into creative practices, its application in public art—particularly in urban sculpture—raises fundamental questions regarding aesthetic cognition, emotional engagement, and artistic acceptance. This study proposes and empirically tests a conceptual model to explain how general audiences perceive and evaluate AIGC-generated urban sculptures. Drawing upon Leder et al.’s aesthetic appreciation framework and theories of human–AI trust, we develop a structural equation model (SEM) comprising seven latent constructs: visual aesthetic features, cognitive mastery, emotional arousal, perceived artistic value, trust in AIGC, artistic acceptance intention, and familiarity control. A total of 24 AI-generated sculpture stimuli were produced using Midjourney v6 and evaluated along five aesthetic dimensions through expert visual content analysis. Questionnaire data were collected from 326 respondents across sculpture parks, art plazas, and university campuses in China. SEM results reveal that both cognitive mastery and emotional arousal significantly mediate the relationship between aesthetic features and perceived artistic value. Moreover, trust in AIGC and perceived artistic value jointly predict acceptance intentions, highlighting the intertwined roles of perceptual, affective, and attitudinal factors in the legitimation of AI-generated art. This research extends classical aesthetic theory to non-human creative contexts and provides practical implications for the design, deployment, and public communication of algorithmically generated urban artworks. By demonstrating that audiences can cognitively and emotionally resonate with AI-generated sculptures—contingent on visual coherence, symbolic richness, and technological trust—this study offers a novel empirical foundation for future investigations into the cultural and spatial integration of artificial creativity. However, the ecological validity of the study is inherently limited, as the stimuli consisted of digital renderings rather than physical public sculptures. Therefore, the findings represent preliminary insights into audience responses to conceptual AIGC artworks.

## Introduction

In recent years, artificial intelligence-generated content (AIGC) has emerged as a transformative force in creative industries, enabling machines to participate in ideation, form generation, and even cultural production. Among various creative domains, the integration of AIGC into public art—particularly urban sculpture—has drawn increasing attention. Its potential to transform aesthetic expression, design methodology, and participatory experience in public spaces makes this integration especially significant [1]. Leveraging powerful generative models such as Midjourney, DALL·E, and Stable Diffusion, artists and designers are now able to produce highly diverse and complex visual forms through text-to-image prompts and algorithmic creativity [2].

Urban sculptures serve not only as spatial markers and aesthetic embellishments but also as embodiments of collective memory and cultural narratives. Traditionally, their creation has relied heavily on human intuition, symbolic intention, and craftsmanship [3]. However, the involvement of AIGC raises pressing questions: Can algorithm-generated sculptures evoke genuine aesthetic appreciation? How do viewers cognitively and emotionally engage with artworks not authored by humans? More fundamentally, are such works perceived as authentic or legitimate artistic expressions [4]?

Despite growing technical advances, the public perception and artistic acceptance of AI-generated urban sculptures remain underexplored. While prior studies have investigated AI in digital art, architecture, and design, few have systematically examined how lay users cognitively process and emotionally respond to AIGC public artworks—especially in the context of complex and symbolic artifacts like sculptures situated in civic environments [5,6].

To address this gap, the present study aims to investigate the mechanisms underlying aesthetic cognition and artistic acceptance of AIGC urban sculptures. Drawing on Leder et al.’s (2004) model of aesthetic appreciation and integrating constructs from technology acceptance and trust in AI literature, we develop a conceptual framework that captures how perceptual, emotional, and evaluative factors shape users’ judgments and intentions [7]. We empirically validate the framework through structural equation modeling (SEM) based on questionnaire data, and complement it with visual content analysis of AI-generated sculpture concepts. By combining cognitive modeling and visual analysis, this study provides a multifaceted understanding of how algorithmically created urban art is experienced, interpreted, and accepted.

The contributions of this research are threefold. First, it extends aesthetic cognition theory to the emerging domain of AI-generated public sculpture, offering insights into how audiences perceive and evaluate non-human-created art. Second, it introduces and validates a hybrid model combining psychological appraisal and acceptance mechanisms. Third, it offers practical implications for designers, urban planners, and policymakers considering the integration of AIGC in future public art initiatives.

Before proceeding, it is essential to acknowledge a key limitation that frames the scope of this study. At present, AIGC public sculptures are rarely realized as physical installations, making it necessary to investigate audience responses using two-dimensional digital concept renderings rather than embodied urban artworks. Consequently, the study should be understood as an early, exploratory examination of how the public cognitively and affectively engages with conceptual AIGC sculptures. There remains a substantial gap between viewing digital images and experiencing public art in situ—where materiality, spatial scale, and environmental context play critical roles—and this distinction is explicitly recognized in the interpretation of our findings.

## Literature foundation and conceptual model development

### AIGC and its emerging role in urban sculpture

AIGC has rapidly gained traction as a creative technology driving innovation in public and environmental art. Recent empirical studies reveal that integrating AIGC into public art practice yields both technical efficiency and enhanced aesthetic engagement, particularly when applied to urban sculpture.

Juan Li (2025) investigated AI-generated artwork in environmental public art, introducing a generative AI technique optimized through Gestalt visual perception principles. In an experiment involving fifty participants, Li reported that perceived quality, social impact, and willingness to accept AI‑generated public art scored highly—suggesting that generative AI can produce visually compelling designs aligned with public expectations [8]. The study also identified concerns regarding economic maintenance costs and technological reliability risks, underscoring that aesthetic innovation must be balanced with practical feasibility to ensure public acceptance.

Moving beyond purely visual aesthetics, Yu Wu (2025) offers a comprehensive framework situating AI art within the context of smart cities. This framework—“technology empowerment–scene reconstruction–ecological collaboration”—demonstrates how algorithmic style transfer, data‑driven creation, and real‑time interactivity reshape urban public art narratives [9]. The study emphasizes the dynamic role of AI in revitalizing cultural heritage and enabling citizen participatory creation while cautioning about ethical, aesthetic, and data‑governance challenges that must be addressed.

Yan Guangyou (2025) explored the synergy between AIGC and virtual reality (VR) technologies in urban public art, based on pilot case studies from cities such as Kyoto and Shenzhen. In this study, participants utilized VR headsets to navigate immersive, AIGC-generated urban environments, enabling real‑time interaction with evolving virtual landscapes. This integration shifts the role of art from static display to an embodied interactive experience, fostering augmented participatory loops among the public [10]. Methodologically, the study employed spatial occupancy heat maps to quantify user movement patterns and dwelling hotspots within the virtual space, while semi-structured interviews were conducted to qualitatively assess how these shared virtual experiences fostered a sense of community cohesion and reinforced local cultural identity.

Moreover, Charis Avlonitou and Eirini Papadaki (2025) critically examined AI as a creative tool across artistic disciplines, including sculpture [11]. Focusing on works by prominent digital artists such as Refik Anadol, this article discusses how generative AI transcends traditional aesthetic norms by blending data visualization with expressive form, while also raising questions about authorship, algorithmic opacity, and the balance between innovation and human creativity.

Across these studies, there is consensus that AIGC offers transformative potential: it expedites creative workflows, enables data‑informed urban design, and invites public participation. However, most scholarship remains limited to conceptual frameworks or experimental techniques, and few studies exclusively address the public aesthetic reception of physical AI‑generated sculptures situated in urban public spaces.

In summary, existing literature substantiates AIGC's relevance to environmental and public art contexts. It outlines both technological possibilities and aesthetic–social dynamics. Nevertheless, there is a clear research gap regarding how AI‑generated design features influence public perception, emotional arousal, trust in AI systems, and acceptance of AI‑based urban sculptures. This gap motivates the current investigation into the mechanisms of aesthetic cognition and artistic acceptance in AIGC‑generated urban sculpture.

### Aesthetic cognition theory: Understanding viewer experience

Helmut Leder et al.’s seminal information‑processing model of aesthetic appreciation introduces a five‑stage framework that has become foundational in empirical aesthetics. According to their theory, aesthetic experience unfolds sequentially through perceptual analysis, implicit memory integration, explicit classification, cognitive mastering, and evaluation, culminating in two distinct outputs: aesthetic judgment and aesthetic emotion.

The perceptual analysis stage involves rapid sensory processing of formal features such as symmetry, complexity, and color contrast. Next, implicit memory integration uses familiarity and prototypicality to modulate early experience. As the process advances, explicit classification engages with interpretive categories including style, genre, and semantic content. In cognitive mastering, meaning is negotiated through layered interpretations—art‑specific, self‑referential, and contextual—and this stage is deeply linked with knowledge structures [12]. Finally, evaluation provides a hedonic-affective assessment, feeding back to refine earlier stages and shaping both felt emotion and explicit aesthetic judgments [13].

Since its publication, Leder's model has spurred empirical work elucidating how viewer expertise, art knowledge, and contextual priming influence aesthetic outcomes. For instance, Belke et al. (2006) demonstrated that providing stylistic information significantly enhanced aesthetic judgments of abstract paintings, suggesting that explicit classification and cognitive mastering are critical for appreciation of ambiguous or novel artworks [14].

Pelowski and Akiba (2011) proposed an alternative five‑stage schema emphasizing disruption and subsequent metacognitive transformation [15], theorizing how art can provoke insight, self‑reflection, and transformative aesthetic experiences beyond conventional mastery processes.

More recently, Christensen, Cardillo, and Chatterjee (2023) reviewed aesthetic cognitivism in Psychology of Aesthetics, Creativity, and the Arts, considering whether art promotes knowledge acquisition [16]. They assert that viewer engagement—including states of curiosity, confusion, and insight—is essential for aesthetic experience to yield understanding and shift cognitive frameworks, resonating with Leder's cognitive mastering concept.

In addition, Pelowski et al. (2016) extended Leder's model into the Vienna Integrated Model of top‑down and bottom‑up processes (VIMAP), incorporating notions such as flow, insight moments, and emotional resonance [17]. They further integrate the aesthetic triad framework—sensory‑motor, emotion‑valuation, meaning‑knowledge systems—to explain how aesthetic experience emerges from interactions between perceptual, affective, and cognitive components.

Empirical studies using ERP and eye‑tracking methods also support the sequential stages of Leder et al.’s model. For example, Höfel & Jacobsen (2007) demonstrated that early perceptual signatures correspond to the perceptual analysis stage, whereas later neural indices reflect explicit classification and evaluation [18]. This temporal evidence reinforces the model's neurocognitive plausibility.

Collectively, these works affirm that aesthetic experience is neither purely perceptual nor exclusively emotional, but arises from dynamic interplay among formal sensory input, memory, cultural knowledge, interpretive effort, and affective evaluation. The five‑stage model remains highly applicable to novel stimuli such as AI‑generated urban sculptures, wherein unfamiliar visual features (e.g., novel form, symmetry anomalies, semantic ambiguity) invite deep cognitive engagement. As such, this framework provides a robust theoretical foundation for modeling how viewers process and respond to AIGC‑generated artworks in public spaces. To empirically operationalize this framework, the present study employed a survey-based evaluation task. Participants were presented with a series of AI-generated conceptual urban sculptures and were tasked with processing these visual stimuli to report their cognitive mastery, emotional arousal, and subsequent acceptance intentions via a structured questionnaire. This specific experimental design allows us to directly map the theoretical stages of aesthetic processing onto observable audience responses.

### Research hypotheses and conceptual framework

The increasing integration of artificial intelligence into the domain of public art, particularly through generative models capable of producing urban and landscape sculptures, presents an urgent need to understand how such creations are cognitively and emotionally processed by human audiences, and under what conditions they are accepted as legitimate art forms. Building on theories of aesthetic cognition (Leder et al., 2004), affective response, and human–AI interaction, we propose a conceptual framework that models the perceptual, cognitive, and attitudinal mechanisms driving artistic acceptance of AIGC-generated public sculpture.

The framework centers on two theoretical premises:(1) that aesthetic evaluation is both a cognitive and emotional process triggered by perceptual input; (2) that audience acceptance of AI-generated art is influenced by trust in the AI's creative capacity, alongside the perceived aesthetic value of its output.

#### Mathematical representation of the model.

To formalize the theoretical relationships among latent variables proposed in our conceptual framework, we present a recursive structural model composed of three stages: (1) perceptual input, (2) cognitive and emotional processing, and (3) evaluative outcome. These stages reflect the sequential transformation of aesthetic stimuli into acceptance intentions through cognitive and attitudinal mechanisms.

1 Perceptual Input to Cognition and Emotion:

$$CM=\gamma_{\text{1}}\cdot \psi_{\text{1}}(VAF)+\epsilon_{\text{1}}$$ (1)

$$EA=\gamma_{\text{2}}\cdot \psi_{\text{2}}(VAF)+\epsilon_{\text{2}}$$ (2)

Where: *VAF* is a vector representing the perceived visual aesthetic features of the sculpture (e.g., symmetry, novelty, complexity, balance, and meaningfulness). *CM* denotes Cognitive Mastery, which refers to the viewer's ability to interpret, understand, or conceptually process the artwork. *EA* is emotional arousal, capturing the intensity of emotional reactions.$\psi_{\text{1}}$ and $\psi_{\text{2}}$ are functions transforming perceptual input into psychological responses.γ_1_,γ_2_ are structural path coefficients $\epsilon_{\text{1}}$, $\epsilon_{\text{2}}$ are residual error terms [19].

These expressions reflect the assumption that different aspects of visual perception activate both cognitive interpretation and emotional engagement.

2 Cognition and Emotion to Artistic Value

$$PAV=\lambda_{\text{1}}\cdot CM+\lambda_{\text{2}}\cdot EA+\epsilon_{\text{3}}$$ (3)

Where: *PAV* denotes perceived artistic value, referring to the viewer's judgment of the artwork's quality, meaning, or originality. $\lambda_{\text{1}}$, $\lambda_{\text{2}}$ are path coefficients indicating the influence of cognitive mastery and emotional arousal on perceived value. $\epsilon_{\text{3}}$ captures individual differences in value judgments not explained by cognition or emotion [20].

This equation reflects the idea that aesthetic value is co-constructed by meaning (cognition) and feeling (emotion). The stronger a person's understanding and emotional engagement, the higher the likelihood of valuing the sculpture artistically [21].

3 Artistic Acceptance Intention as Final Outcome

$$AAI=\beta_{\text{1}}\cdot PAV+\beta_{\text{2}}\cdot TA+\beta_{\text{3}}\cdot FC+\epsilon_{\text{4}}$$ (4)

Where: *AAI* denotes Artistic Acceptance Intention, referring to the degree to which a viewer is willing to accept the AIGC-generated sculpture as a legitimate form of public art. *TA* denotes Trust in AIGC, reflecting belief in the AI system’s capability to produce authentic, creative, and culturally meaningful content. *FC* denotes Familiarity Control, representing participants’ prior exposure to AI-generated artworks and public sculpture, included as a control variable to account for baseline differences in acceptance intention. *β₁*, *β₂*, and *β₃* are structural path coefficients, and ε₄ is the residual error term [22,23].

This final equation integrates both evaluative appraisals (value) and attitudinal predispositions (trust) into the decision to accept or reject the artistic legitimacy of an AIGC-produced sculpture [24].

#### Hypotheses and conceptual framework.

Based on the structural model defined above, we propose a set of research hypotheses that describe the directional relationships among the key constructs involved in the aesthetic evaluation and acceptance of AIGC-generated urban sculptures.

The model assumes that visual aesthetic features influence both cognitive mastery and emotional arousal, which in turn affect the perceived artistic value of the sculpture. Perceived artistic value and trust in AIGC jointly contribute to the viewer's intention to accept the sculpture as legitimate public art.

Accordingly, we formulate the following hypotheses: H1: Visual aesthetic features positively influence cognitive mastery. H2: Visual aesthetic features positively influence emotional arousal. H3: Cognitive mastery positively influences perceived artistic value. H4: Emotional arousal positively influences perceived artistic value. H5: Perceived artistic value positively influences artistic acceptance intention. H6: Trust in AIGC positively influences artistic acceptance intention. H7: Familiarity Control positively influences Artistic Acceptance Intention.

**Fig 1:**
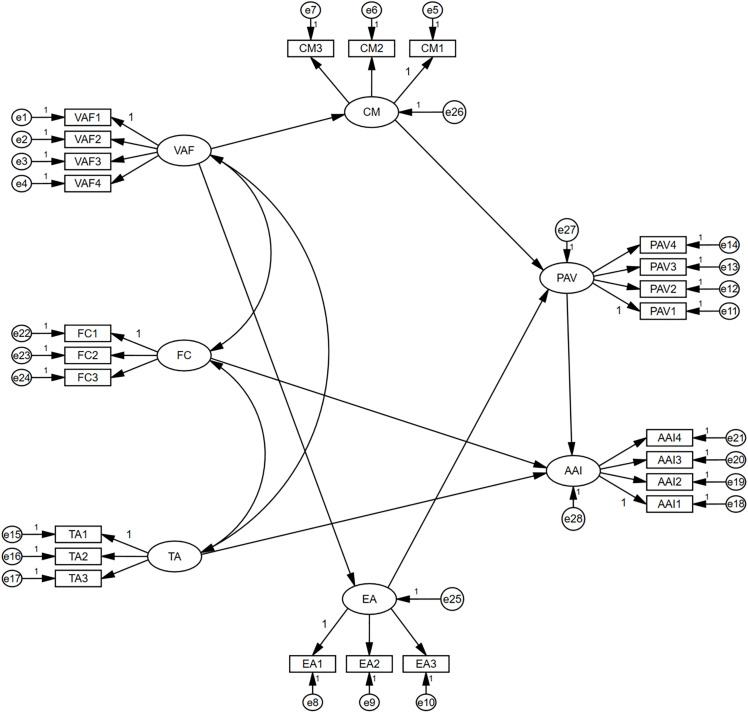
Conceptual framework illustrating the hypothesized relationships among visual aesthetics, cognition, emotion, trust, and artistic acceptance intention. **Note:** VAF = Visual Aesthetic Features; CM = Cognitive Mastery; EA = Emotional Arousal; PAV = Perceived Artistic Value; TA = Trust in AIGC; FC = Familiarity Control; AAI = Artistic Acceptance Intention.

To visually illustrate the hypothesized structure, Fig 1 presents the conceptual framework. All latent variables are represented as nodes, and hypothesized causal paths are indicated with directional arrows. Each path corresponds to a specific hypothesis (H1–H7), which will be empirically tested using structural equation modeling.

For clarity and reference during model estimation, Table 1 summarizes the mapping between each hypothesis, the corresponding structural path, and its associated equation:

**Table 1:** Hypotheses, Path Relationships, and Equation Mapping.

Hypothesis	Structural Path	Equation Reference	Expected Direction
H1	Visual features → Cognition	$CM=\gamma_{\text{1}}\cdot \psi_{\text{1}}(VAF)+\epsilon_{\text{1}}$	Positive (+)
H2	Visual features → Emotion	$EA=\gamma_{\text{2}}\cdot \psi_{\text{2}}(VAF)+\epsilon_{\text{2}}$	Positive (+)
H3	Cognition → Artistic value	$PAV=\lambda_{\text{1}}\cdot CM+\lambda_{\text{2}}\cdot EA+\epsilon_{\text{3}}$	Positive (+)
H4	Emotion → Artistic value	Same as above	Positive (+)
H5	Artistic value → Acceptance	$AAI=\beta_{\text{1}}\cdot PAV+\beta_{\text{2}}\cdot TA+\beta_{\text{3}}\cdot FC+\epsilon_{\text{4}}$	Positive (+)
H6	Trust → Acceptance	Same as above	Positive (+)
H7	Familiarity Control → Acceptance	Same as above	Positive (+)

This integrated framework provides the theoretical and analytical foundation for empirical testing in the subsequent methodology section. Each hypothesis is operationalized as a testable relationship within the structural model and contributes to understanding how perceptual, cognitive, emotional, and attitudinal factors jointly shape human responses to AI-generated public art [25].

## Methodology

### Research design overview

To systematically investigate the perceptual, cognitive, and attitudinal mechanisms underlying public responses to AIGC-generated urban sculptures, this study adopts a quantitative research design that combines visual content analysis with structural equation modeling (SEM). The design ensures theoretical rigor and methodological transparency across stimulus generation, instrument development, data collection, and model testing.

The research process is structured in the following three phases:

(1) Visual Stimuli Generation Using Midjourney

We employed Midjourney v6, a state-of-the-art generative AI platform, to produce a curated set of digital urban sculpture concepts. A series of prompt engineering techniques were implemented to generate diverse and semantically rich sculptures varying in visual complexity, novelty, symmetry, and thematic abstraction. These images were created to reflect a range of design expressions plausible for civic installation and were subsequently reviewed by five experts in public art and computational aesthetics to ensure visual realism and ecological validity [26].

The resulting set of AI-generated sculpture images was used as the visual stimulus material for participant evaluation and content analysis. Visual features were later coded along predefined aesthetic dimensions to support the interpretation of perceptual input in the SEM model.

(2) Questionnaire Design Based on Aesthetic Cognition and Acceptance Theory

Guided by Leder et al.’s (2004) five-stage aesthetic processing model and supplemented by constructs from the literature on emotional arousal, perceived artistic value, and trust in AI, we developed a comprehensive measurement instrument. Each latent construct was operationalized using multiple items on a 5-point Likert scale, adapted from prior validated scales to reflect the context of AIGC-generated public art.

A pilot test involving 50 participants was conducted to assess item clarity, content validity, and internal consistency [27]. Based on feedback and item-level statistics (e.g., corrected item-total correlation, Cronbach's α), the questionnaire was revised prior to full-scale deployment.

(3) Data Collection and Structural Model Estimation

A total of 400 printed questionnaires were distributed via offline field surveys in urban public spaces across three cities in China, including sculpture parks, art exhibition plazas, and university campuses with design-related disciplines. These locations were strategically selected to ensure that respondents had prior exposure to public art and visual design stimuli, enhancing ecological validity. The inclusion criteria targeted general adult viewers aged 18–65, without requiring formal training in art or AI.

Participants were randomly assigned to view a subset of the AI-generated sculpture images before completing the questionnaire. The data were collected anonymously and voluntarily, following institutional ethical approval.

Data entry and preliminary analysis were conducted using SPSS 27, including descriptive statistics, reliability tests, and exploratory factor analysis (EFA). Subsequently, AMOS 24.0 was used to perform confirmatory factor analysis (CFA) and structural equation modeling. Model evaluation followed established SEM guidelines, with key fit indices reported including χ²/df, CFI, TLI, RMSEA, and SRMR [28].

By integrating AI-generated stimuli, aesthetic cognition theory, and rigorous empirical modeling, this research design provides a robust framework for understanding how the general public perceives and accepts AI-generated urban sculptures. The mixed use of visual content and psychometric modeling ensures high external and internal validity, contributing both to theoretical development in empirical aesthetics and to practical design evaluation in AI-assisted public art.

### Visual stimuli creation and content analysis

#### AIGC tools and prompt strategy.

To generate visual stimuli that reflect the diverse expressive possibilities of AI-generated public art, this study utilized Midjourney v6, a leading text-to-image diffusion model known for its advanced control over texture, form, lighting, and composition. Midjourney was selected for its superior capabilities in rendering three-dimensional, sculpture-like structures with high semantic and material fidelity—attributes critical for simulating the look and feel of real-world urban installations.

A set of 60 sculpture concepts were initially generated using carefully designed prompt instructions. To ensure representational diversity and alignment with public art contexts, we implemented a systematic prompt engineering strategy, wherein textual inputs were varied across key design dimensions [29]. These dimensions included material type, spatial scale, formal articulation, symbolic content, and hybrid complexity. Example prompt structures are provided in Table 2, illustrating how linguistic choices can shape visual outputs across material realism, thematic richness, and compositional balance.

**Table 2:** Categories of Prompt Strategies and Representative Examples Used in Midjourney Generation.

Prompt Type	Example Prompt	Design Focus
Material-Oriented	“A public sculpture made of polished bronze and textured granite, reflecting sunlight with high contrast materials.”	Surface realism and material texture
Scale & Composition	“A large-scale modular urban sculpture composed of interconnected vertical slabs, designed for a civic plaza.”	Structural massing and spatial context
Formal Elements	“A twisting sculpture with bilateral symmetry, fine curvature, and perforated surface texture, 3m tall.”	Visual rhythm, balance, and detail
Thematic Content	“A contemporary sculpture inspired by marine biodiversity, abstract coral forms rendered in matte white.”	Narrative, symbolism, and cultural themes

After generation, all 60 images were independently reviewed by two domain experts with backgrounds in computational aesthetics and public sculpture design. Evaluation criteria included: Visual realism (e.g., plausible shading, materials, depth), Semantic clarity (e.g., clear motifs or thematic expression), Installability (e.g., plausible scale and physical feasibility).

**Fig 2:**
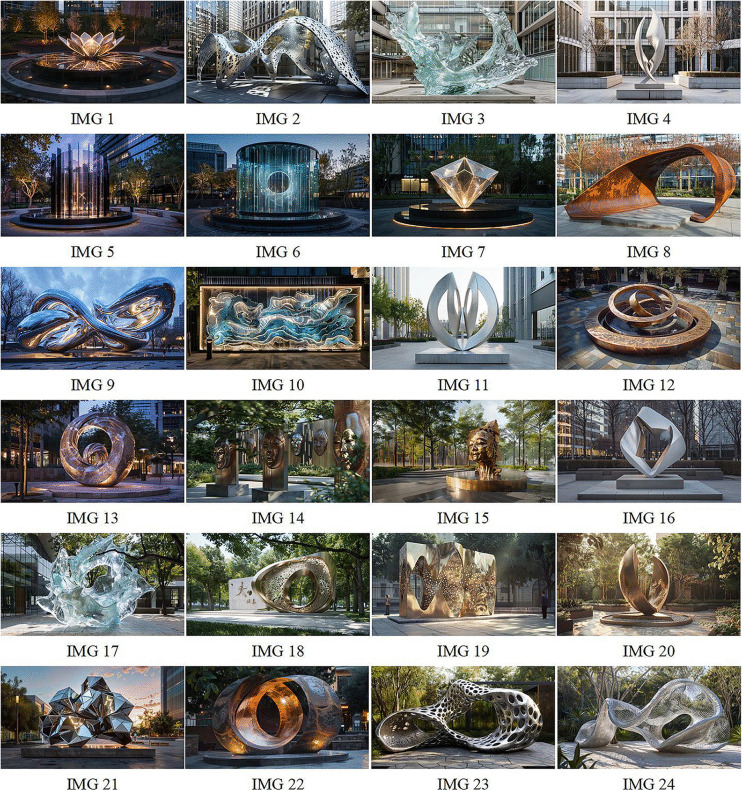
Representative set of 24 AIGC-generated urban sculpture concepts created using Midjourney v6.

Based on these criteria, a final set of 24 images was selected as the official visual stimuli. These images are shown in Fig 2, which demonstrates the stylistic and semantic diversity achieved through prompt variation. The selected images collectively represent a wide range of public art aesthetics—from minimalist monoliths to symbolically charged, biomorphic structures.

The prompt engineering approach employed here goes beyond mere image generation; it serves as a parametric visual programming method in which textual levers control conceptual and perceptual outcomes. This method enabled the construction of stimuli that are not only visually compelling but also theoretically anchored in the domains of urban art, material culture, and AI-human co-creativity [30].

#### Visual analysis dimensions.

To quantitatively assess the aesthetic characteristics of the AIGC-generated sculpture stimuli, we employed a structured visual content analysis framework grounded in theories of empirical aesthetics, perceptual psychology, and public art design. The goal was to capture key perceptual variables that influence cognitive processing and aesthetic judgment in viewer responses.

Each of the 24 selected sculpture images was evaluated along five core dimensions, defined as follows:

Visual Complexity: Refers to the degree of visual intricacy, including the number of distinct elements, compositional layering, and surface variation. This metric reflects cognitive load and is often linked to delayed but deeper appreciation.

Symmetry and Balance: Captures both formal symmetry (bilateral or radial) and the equilibrium of visual weight across the sculpture. This dimension influences perceptual fluency and overall harmony.

Novelty: Measures how much the sculpture diverges from familiar or prototypical forms. Novel designs are associated with higher curiosity, interpretive depth, and emotional arousal.

Perceived Meaning: Represents the semantic and symbolic richness of the sculpture—its metaphorical references, emotional resonance, or cultural narratives. High meaningfulness supports sustained viewer engagement and reflective interpretation.

Material Plausibility: Evaluates whether the depicted materials (e.g., reflectivity, density, translucency) are realistic and feasible within public space installations. This ensures ecological validity and enhances viewer trust in authenticity.

#### Coding procedure and inter-rater reliability.

To ensure the objectivity and reliability of the visual assessment, a rigorous coding protocol was implemented. The panel of coders consisted of five domain experts (three professors of public art and two senior computational designers), none of whom were involved in the initial image generation process.

The coding procedure followed a blind review format:

1. Training: Coders were briefed on the operational definitions of the five aesthetic dimensions (as defined in Section 3.2.2) to ensure conceptual alignment.
2. Independent Scoring: The 24 selected stimuli were presented in random order. Coders rated each image on the five dimensions using a 5-point Likert scale (1 = Low, 5 = High).
3. Reliability Test: Inter-rater reliability was assessed using the Intraclass Correlation Coefficient (ICC) (Two-way mixed, consistency type). The resulting average ICC was 0.85 (95% CI [0.81, 0.89]), indicating “excellent” agreement among raters.
4. Data Aggregation: The final scores for each dimension were derived by averaging the ratings across the five coders to minimize individual subjective bias.

### Questionnaire design

#### Construct operationalization.

Seven latent variables were measured in this study, each corresponding to a core component of the proposed conceptual model. All constructs were operationalized using multi-item reflective indicators, measured on a 5-point Likert scale ranging from 1 = strongly disagree to 5 = strongly agree. The constructs and their theoretical bases are described as follows:

Visual Aesthetic Features (VAF): This exogenous construct captures participants’ perception of formal visual elements of the sculptures, including symmetry, novelty, complexity, balance, and meaningfulness. It is conceptually linked to the perceptual analysis and explicit classification stages in Leder's model.

Cognitive Mastery (CM): Refers to the degree to which participants believe they can understand, interpret, or intellectually engage with the artwork. Derived from the cognitive mastering stage in aesthetic processing models.

Emotional Arousal (EA): Captures the intensity of affective response evoked by the sculptures, such as feelings of wonder, surprise, or inspiration. It draws on affective dimensions in both Leder's model and appraisal theory.

Perceived Artistic Value (PAV): Reflects judgments about the overall artistic quality, originality, and meaningfulness of the sculpture. It is positioned as a mediating variable between cognition/emotion and acceptance.

Trust in AIGC (TA): Indicates the degree of trust participants place in AI-generated content as authentic, ethical, and creatively competent. This construct is rooted in the literature on human–AI interaction and creative automation trustworthiness.

Artistic Acceptance Intention (AAI): Measures participants’ willingness to accept the AI-generated sculpture as a valid form of public art. It reflects behavioral intention based on cognitive and attitudinal assessments.

Familiarity Control (FC): A control variable was included to measure participants’ prior familiarity with AI-generated art and public sculpture to account for possible bias in interpretation.

Each construct was informed by prior validated measurement models in the domains of aesthetics, AI perception, and user acceptance. Items were adapted linguistically to reflect the specific context of viewing algorithmically generated urban sculptures.

#### Scale development and operationalization.

To ensure rigorous construct operationalization and strong theoretical grounding, the measurement instrument was developed based on established frameworks in empirical aesthetics, psychology of art, and human–AI interaction research. The final instrument comprised seven latent constructs measured using a total of 24 reflective indicators. All items were rated on a 5-point Likert scale (1 = strongly disagree, 5 = strongly agree), with higher scores indicating stronger agreement [31].

The scale items were adapted from previously validated instruments and reworded to fit the specific context of AIGC-generated public sculpture while preserving their original conceptual meanings. Table 3 presents the detailed operationalization of each construct, including its operational definition, original academic sources, and the full set of measurement items used in the survey.

**Table 3:** Scale Operationalization: Definitions, Sources, and Measurement Items.

Construct	Operational Definition & Theoretical Source	Measurement Items
Visual Aesthetic Features (VAF)	The viewer’s perception of the formal visual properties of the stimuli, focusing on structural coherence and novelty. Based on the Perceptual Analysis stage of aesthetic processing models. Source: Adapted from Leder et al. (2004) [7] and Pelowski et al. (2016) [17].	The sculpture appears visually rich and complex.
		The composition of the sculpture feels balanced and harmonious.
		The sculpture introduces a novel and unconventional visual form.
		The sculpture conveys symbolic or metaphorical meaning through its design.
Cognitive Mastery (CM)	The extent to which the viewer feels capable of understanding, interpreting, or conceptually engaging with the artwork’s intent. Source: Adapted from Leder et al. (2004) [7] and Belke et al. (2006) [14].	I can grasp the underlying idea or concept behind the sculpture.
		I find the design intellectually engaging and worth interpreting.
		I feel confident in interpreting the artistic intent of this sculpture.
Emotional Arousal (EA)	The intensity of the affective response (e.g., curiosity, wonder, stimulation) evoked by the visual stimuli. Source: Adapted from Silvia (2005) [19] and Grassini & Koivisto (2024) [25].	This sculpture evokes a strong emotional reaction in me.
		I feel a sense of curiosity or wonder when viewing the sculpture.
		The sculpture stimulates my imagination and feelings.
Perceived Artistic Value (PAV)	The viewer’s evaluative judgment regarding the artwork's creativity, meaningfulness, and legitimacy as art. Source: Adapted from Schindler et al. (2012) [21] and Leder et al. (2004) [7].	This sculpture demonstrates high artistic creativity and originality.
		I perceive this sculpture as an expressive and meaningful artwork.
		The sculpture would be appropriate for display in public art venues.
		This work deserves recognition as a legitimate form of art.
Trust in AIGC (TA)	The belief in the AI system's capability to produce content that is aesthetically valuable, culturally meaningful, and authentic. Source: Adapted from Choung et al. (2023) [31] and Nazrin et al. (2025) [22].	I trust that AI can create artistically valuable public sculptures.
		I believe AI-generated artworks can carry cultural and symbolic meaning.
		I think AI systems are capable of producing aesthetically appealing art.
Artistic Acceptance Intention (AAI)	The behavioral intention to accept, support, and recommend the integration of AI-generated sculptures in public urban spaces. Source: Adapted from Nazrin et al. (2025) [22] and Venkatesh et al. (2003) [32].	I would accept this sculpture as a valid form of public art.
		I support exhibiting AI-generated sculptures in public urban spaces.
		I am open to experiencing art created by artificial intelligence.
		I would recommend this type of sculpture for inclusion in city spaces.
Familiarity Control (FC)	A control variable measuring the viewer's prior exposure to and knowledge of AI technologies and public art. Source: Adapted from Grassini & Koivisto (2024) [25] and Liu et al. (2024) [4].	I am familiar with AI-generated images or artworks.
		I have seen public sculptures or installations in urban spaces.
		I actively follow developments in AI and digital creativity.

### Data collection procedure

To ensure empirical robustness and contextual relevance, data collection was conducted through an offline questionnaire-based field survey targeting individuals with direct exposure to urban public art. The procedure was designed to maximize ecological validity while maintaining sample diversity and methodological rigor.

The survey adopted a purposive sampling strategy to reach non-expert yet visually literate respondents who were likely to encounter and evaluate public sculpture in real-world settings. Three types of urban public spaces were selected across three representative Chinese cities to capture regional and demographic variability: 1. Urban sculpture parks, known for hosting permanent outdoor installations; 2. Public art plazas, typically located in commercial or cultural districts; 3. University campuses with strong art, design, or architecture faculties.

These sites were selected to ensure respondents had prior or ongoing exposure to visual design, cultural symbols, and public art installations, thereby enhancing the validity of their perceptual and attitudinal judgments.

Participants were recruited onsite through face-to-face intercept sampling. Inclusion criteria were: 1. Adults aged 18–65 years; 2. No formal training in art or AI required; 3. Ability to complete a visually anchored questionnaire in Mandarin.

To reduce selection bias, both weekdays and weekends were included, and data were collected across different time periods of the day (morning, afternoon, early evening).

A total of 400 printed questionnaires were distributed over a four-week period in September 2025. Prior to answering, each participant was shown a curated subset of 3–4 AIGC-generated urban sculpture images (selected randomly from the full image set used in Fig 2). These images were printed in high resolution to preserve their visual details and were briefly introduced as “AI-generated conceptual sculptures intended for public spaces.”

Participants were instructed to review the images carefully before proceeding to complete the questionnaire, which comprised: Demographic questions (age, gender, education, occupation); Familiarity check items with AI and public art (FC); 24 items corresponding to the six latent constructs in the conceptual model (VAF, CM, EA, PAV, TA, AAI), using 5-point Likert scales.

All responses were recorded anonymously and voluntarily. Survey administrators were trained to remain neutral and non-directive during the process. To improve data reliability, participants were encouraged to ask for clarification on item meanings but were not provided with interpretive guidance [33].

The study was approved by the institutional research ethics committee of the authors’ affiliated university. All participants were informed of the study's academic purpose, assured of the confidentiality of their responses, and provided with the option to withdraw at any time. No identifying information was collected, and no compensation was offered, minimizing response bias.

Of the 400 questionnaires distributed, 326 were returned and deemed valid after manual screening for completeness and patterned responses (valid response rate: 81.5%). The final sample covered a broad range of age groups, educational backgrounds, and exposure levels to AI-generated content, supporting the generalizability of the findings within the scope of public visual culture.

This study strictly followed all applicable ethical guidelines and regulations regarding human subject research. All procedures were reviewed and approved by the Institutional Research Ethics Committee of Quanzhou Normal University. Participants were informed about the academic purpose of the study, their anonymity was ensured, and informed consent was obtained from all respondents prior to participation. No personal identifiable information was collected, and participation was entirely voluntary.

### Analytical approach: Structural equation modeling (SEM)

To examine the hypothesized relationships among aesthetic perception, cognitive and emotional processing, and the acceptance of AIGC-generated urban sculptures, this study adopted a Structural Equation Modeling (SEM) approach. SEM was selected due to its ability to estimate complex causal relationships among multiple latent constructs while simultaneously accounting for measurement error.

A two-step SEM procedure (Anderson & Gerbing, 1988) was followed to ensure both the validity of the measurement instrument and the robustness of the structural model [34]:

1 Confirmatory Factor Analysis (CFA) was first performed to assess the psychometric properties of the measurement model, ensuring that all latent constructs exhibited acceptable levels of reliability, convergent validity, and discriminant validity.
2 Upon establishing measurement adequacy, the structural model was estimated to test the hypothesized causal relationships among constructs, using path analysis with latent variables.

This procedure was implemented using SPSS 27 for descriptive statistics and preliminary reliability testing, and AMOS 24.0 for CFA and SEM. Maximum Likelihood Estimation (MLE) was employed as the estimation method, which is robust under conditions of multivariate normality and medium-to-large sample sizes.

## Results

### Descriptive statistics and sample profile

Table 4 summarizes the demographic characteristics of the respondents. The sample included a diverse cross-section of urban residents, students, and professionals with varied exposure to public art and digital technologies.

**Table 4:** Demographic Characteristics of Survey Respondents (N = 326).

Variable	Category	Frequency	Percentage (%)
Gender	Male	168	51.5
	Female	158	48.5
Age Group	18–25	104	31.9
	26–35	89	27.3
	36–45	67	20.6
	46–55	41	12.6
	56–65	25	7.7
Educational Level	High school or below	42	12.9
	Bachelor's degree	192	58.9
	Master's degree or above	92	28.2
Occupation	University student	118	36.2
	Art/design-related professional	71	21.8
	General urban resident	137	42
Exposure to AI Art	Never heard/seen	41	12.6
	Seen online or in media	204	62.6
	Experienced in person	81	24.8

Table 5 provides the descriptive statistics of the seven latent constructs, including means, standard deviations, and reliability estimates (Cronbach's α). All constructs show acceptable levels of internal consistency (α ≥ 0.70), supporting the psychometric adequacy of the measurement model.

**Table 5:** Descriptive Statistics and Reliability Coefficients for Latent Constructs.

Construct	Items	Mean	SD	Cronbach's α
Visual Aesthetic Features (VAF)	4	3.086	0.702	0.845
Cognitive Mastery (CM)	3	3.030	0.662	0.766
Emotional Arousal (EA)	3	3.074	0.715	0.775
Perceived Artistic Value (PAV)	4	3.101	0.623	0.821
Trust in AIGC (TA)	3	3.090	0.696	0.762
Artistic Acceptance Intention (AAI)	4	3.249	0.642	0.838
Familiarity Control (FC)	3	3.172	0.655	0.778

The mean values, ranging from 3.03 to 3.25, indicate a generally moderate and slightly positive orientation across all constructs. Artistic Acceptance Intention (AAI) exhibited the highest mean score (M = 3.249), suggesting that participants were broadly receptive to the integration of AI-generated artworks into public spaces. Perceived Artistic Value (PAV; M = 3.101) and Visual Aesthetic Features (VAF; M = 3.086) also scored above the neutral midpoint, reflecting public recognition of artistic merit and visual appeal in the generated sculptures.

Emotional Arousal (EA) recorded a mean of 3.074, indicating a moderate level of affective engagement. This suggests that while the stimuli were not emotionally neutral, they did not elicit strong emotional reactions, which is consistent with responses to novel or unfamiliar aesthetic content. Cognitive Mastery (CM) showed the lowest mean value (M = 3.030), implying modest interpretive challenges or conceptual ambiguity in understanding AI-generated forms, a pattern commonly observed in studies of algorithmically generated aesthetics.

Trust in AIGC (TA) demonstrated a moderate mean score (M = 3.090), indicating a cautious but present level of confidence in non-human creative agents. Familiarity Control (FC) yielded a slightly higher mean (M = 3.172), suggesting that participants possessed general, rather than expert-level, familiarity with either public art or AIGC technologies. Overall, these descriptive statistics suggest that respondents perceived AIGC-generated sculptures as visually and conceptually acceptable, with moderate emotional engagement and measured trust. This pattern provides a descriptive foundation for the subsequent structural equation modeling analysis [35].

### Measurement model evaluation

Before estimating the structural relationships among latent constructs, a Confirmatory Factor Analysis (CFA) was conducted to assess the validity and reliability of the measurement model. This process involved evaluating the overall model fit, convergent validity, and discriminant validity, ensuring that each construct was adequately captured by its corresponding observed indicators. The analysis was performed using AMOS 24.0, with maximum likelihood estimation.

#### Model fit assessment.

The overall goodness-of-fit of the proposed structural equation model was evaluated using multiple fit indices, as summarized in Table 6. The results indicate that the model demonstrates a satisfactory and statistically acceptable fit to the observed data.

**Table 6:** Goodness-of-Fit Indices for the Structural Equation Model.

Fit Index	Observed Value	Recommended Threshold	Interpretation
Chi-square (χ²)	437.184	—	—
Degrees of Freedom (df)	242	—	—
χ²/df	1.807	< 3.0	Excellent Fit
RMSEA	0.050	< 0.08	Excellent Fit
SRMR	0.076	< 0.08	Acceptable Fit
CFI	0.937	> 0.90	Good Fit
TLI	0.928	> 0.90	Good Fit

Specifically, the chi-square to degrees of freedom ratio (χ²/df) was 1.807, which is well below the recommended threshold of 3.0, indicating a parsimonious model structure. The Root Mean Square Error of Approximation (RMSEA) was 0.050, falling below the commonly accepted cutoff value of 0.08 and suggesting a close approximation between the model-implied and observed covariance matrices. The Standardized Root Mean Square Residual (SRMR) was 0.076, meeting the recommended criterion and indicating an acceptable level of residual discrepancy [36].

In terms of incremental fit indices, both the Comparative Fit Index (CFI = 0.937) and the Tucker–Lewis Index (TLI = 0.928) exceeded the recommended threshold of 0.90, reflecting a strong improvement of the proposed model over the null model. Collectively, these results support the adequacy of the structural equation model and confirm that the hypothesized relationships among the latent constructs are represented in a statistically robust manner.

#### Convergent validity.

Convergent validity was assessed through three indicators for each latent construct: Standardized factor loadings (recommended ≥ 0.60); Composite Reliability (CR) (recommended ≥ 0.70); Average Variance Extracted (AVE) (recommended ≥ 0.50).

All standardized loadings ranged from 0.65 to 0.85, exceeding the minimum threshold and confirming that each indicator strongly represents its respective latent variable. Table 7 presents the results of the convergent validity assessment. As observed, all CR values exceeded 0.70, and AVE values surpassed the 0.50 threshold, indicating adequate convergent validity for all constructs.

**Table 7:** Convergent Validity: Composite Reliability (CR), Average Variance Extracted (AVE), and Indicator Loadings.

Construct	CR	AVE	Lowest Loading
Visual Aesthetic Features (VAF)	0.84	0.57	0.73
Cognitive Mastery (CM)	0.76	0.52	0.66
Emotional Arousal (EA)	0.77	0.53	0.69
Perceived Artistic Value (PAV)	0.82	0.53	0.67
Trust in AIGC (TA)	0.76	0.52	0.67
Artistic Acceptance Intention (AAI)	0.84	0.57	0.65
Familiarity Control (FC)	0.78	0.54	0.69

These results confirm that each latent construct achieves internal consistency reliability and that more than 50% of the variance in its observed indicators is captured by the latent variable, thus establishing strong convergent validity.

#### Discriminant validity.

Discriminant validity was evaluated using the Fornell–Larcker criterion, which requires that the square root of AVE for each construct be greater than its correlations with other constructs.

**Fig 3:**
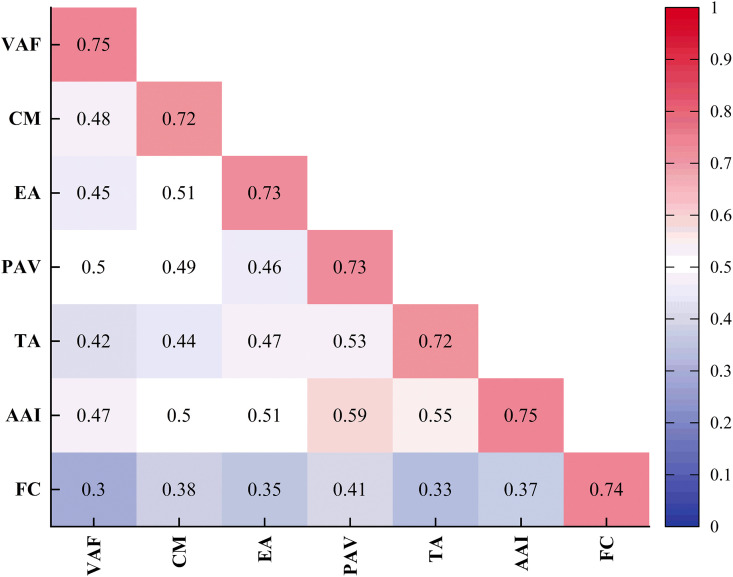
Discriminant Validity: Fornell–Larcker Criterion.

All constructs satisfied this criterion (see Fig 3), indicating clear conceptual distinction between constructs. Furthermore, the HTMT (Heterotrait-Monotrait) ratios for all construct pairs were below 0.85, reinforcing the evidence for discriminant validity [37].

The CFA results support the reliability, unidimensionality, and validity of all constructs in the proposed model. The measurement model demonstrates strong psychometric properties and provides a sound basis for structural model testing in the next phase.

### Structural model results

Table 8 presents the standardized path coefficients, t-values (critical ratios), and significance levels for all hypothesized paths (H1–H7). All hypotheses were supported with statistically significant coefficients (p < 0.001 or p < 0.01), confirming the theoretical relationships posited in the model.

**Table 8:** Structural Equation Modeling Results: Standardized Path Coefficients and Hypothesis Testing.

Pathway	Hypothesis	Estimate (β)	CR (t-value)	p-value	Result
VAF → CM	H1	0.51	6.76	< 0.001	Supported
VAF → EA	H2	0.49	6.60	< 0.001	Supported
CM → PAV	H3	0.34	4.97	< 0.001	Supported
EA → PAV	H4	0.40	5.76	< 0.001	Supported
PAV → AAI	H5	0.29	4.65	< 0.001	Supported
TA → AAI	H6	0.22	3.02	0.003	Supported
FC → AAI	H7	0.28	3.82	< 0.001	Supported

**Fig 4:**
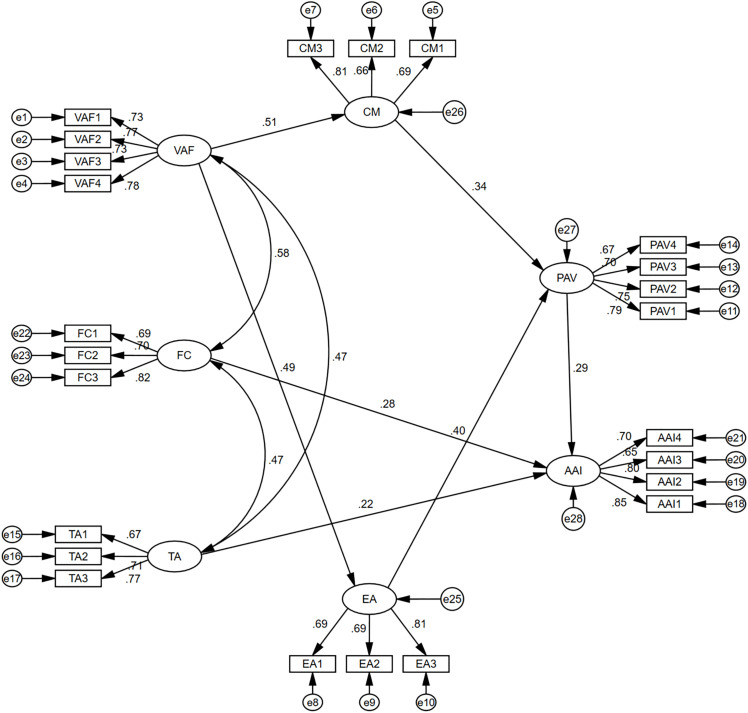
Final structural equation model with standardized path coefficients (N = 326). All paths significant at p < 0.01.

Fig 4 presents the final structural model, including all standardized path coefficients.

Specifically, Visual Aesthetic Features (VAF) exerted a strong positive effect on Cognitive Mastery (CM) (β = 0.51, CR = 6.76, p < 0.001), supporting H1, and also demonstrated a significant direct influence on Emotional Arousal (EA) (β = 0.49, CR = 6.60, p < 0.001), supporting H2. These findings underscore the foundational role of visual aesthetics in shaping both cognitive understanding and emotional responses toward AIGC-generated urban sculptures.

Both Cognitive Mastery and Emotional Arousal were found to positively influence Perceived Artistic Value (PAV). Specifically, CM exhibited a significant effect on PAV (β = 0.34, CR = 4.97, p < 0.001; H3 supported), while EA also showed a strong positive impact on PAV (β = 0.40, CR = 5.76, p < 0.001; H4 supported). These results validate the mediating roles of cognitive and emotional mechanisms in the formation of perceived artistic value.

Furthermore, Perceived Artistic Value significantly predicted Artistic Acceptance Intention (AAI) (β = 0.29, CR = 4.65, p < 0.001), lending support to H5 and indicating that higher perceived artistic value substantially enhances public willingness to accept AIGC-generated sculptures.

In addition, Trust in AIGC (TA) exerted a significant positive effect on AAI (β = 0.22, CR = 3.02, p = 0.003), supporting H6, and highlighting the importance of cognitive trust in generative technologies for public acceptance. Finally, Familiarity Control (FC) also demonstrated a significant, albeit comparatively weaker, influence on AAI (β = 0.28, CR = 3.82, p < 0.001), supporting H7 and suggesting that prior familiarity with related artistic or technological content modestly increases acceptance intention [38].

Overall, the structural model exhibits robust explanatory capability, with all hypothesized paths empirically supported. As illustrated in Fig 4, the significant relationships reveal a comprehensive interplay among visual aesthetics, cognitive appraisal, emotional engagement, and trust-related factors in shaping public acceptance of AI-generated public artworks.

### Findings from visual content analysis

#### Aesthetic attributes of AIGC-generated sculptures.

To deepen the interpretation of participants’ perceptual and attitudinal mechanisms toward AIGC-generated urban sculptures, a systematic visual content analysis was conducted based on 24 image stimuli generated using Midjourney. This analysis complements the SEM results by empirically grounding the theoretical constructs in observable visual features. Specifically, five key aesthetic dimensions were examined: visual complexity, symmetry & balance, novelty, perceived meaning, and material plausibility [39]. Five expert raters independently evaluated all stimuli based on a predefined coding protocol using a 5-point Likert scale.

Visual complexity, reflecting the level of detail, layering, and compositional intricacy, received a moderate average score (M = 3.49). Some sculptures exhibited minimalistic geometries, while others displayed elaborate structures with nested forms and decorative elements. This variability in structural richness provided a foundational basis for perceptual differentiation across stimuli.

Symmetry and balance, indicative of visual coherence and compositional harmony, received the highest overall evaluation (M = 4.11). Most stimuli were purposefully designed with axial or radial symmetry using Midjourney prompt engineering, which likely influenced participants’ cognitive mastery in interpreting the images. This high mean score reinforces the visual stability and order inherent in the stimuli set.

Novelty, as a central component in aesthetic appraisal, achieved a moderately high average rating (M = 3.57). Sculptures featuring unconventional shapes, surprising textures, or hybrid stylistic references (e.g., fusions of biological and mechanical motifs) were rated higher on this dimension. This aligns with their presumed capacity to evoke emotional arousal and artistic curiosity, in support of the SEM pathways.

Perceived meaning (M = 3.55) captured the extent to which sculptures conveyed symbolic depth or narrative potential. Experts evaluated whether the forms suggested metaphorical, historical, or socio-cultural interpretations. Sculptures with recognizable references or evocative postures tended to score higher, reflecting cognitive elaboration during aesthetic processing.

Material plausibility, the extent to which sculptures appeared physically realizable using real-world materials such as stone, metal, or fabric, also received a moderate mean rating (M = 3.53). Stimuli with clear surface texture rendering and realistic shading were evaluated as more constructible in real-world public spaces. This dimension served as a critical visual anchor for evaluating the ecological validity of AIGC-generated works [40].

**Fig 5:**
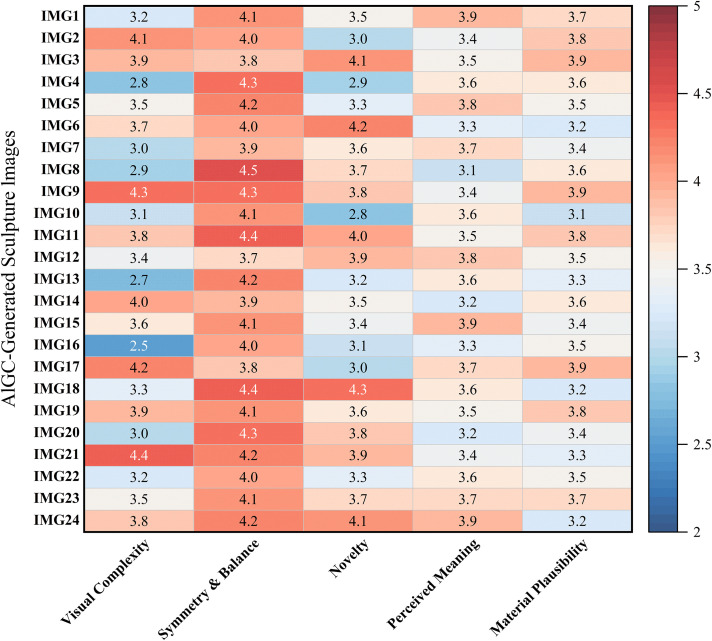
Visual analysis heatmap of 24 AIGC-generated sculpture stimuli rated across five perceptual dimensions.

Fig 5 presents a heatmap of expert ratings across the five aesthetic dimensions, highlighting the perceptual variation embedded within the AIGC-generated sculpture stimuli. Notably, visual complexity and symmetry & balance emerged as dominant perceptual anchors—complexity offered strong differentiation across stimuli due to its varied structural richness (M = 3.49), while symmetry & balance showed the highest mean score (M = 4.11), reflecting perceptual coherence consistently embedded across the dataset.

These two dimensions align with Leder et al.’s aesthetic processing model, which posits that complexity and formal order serve as early-stage perceptual cues that initiate cognitive and emotional engagement. Furthermore, their prominence is consistent with the prompt-engineering strategies employed during image generation, which intentionally emphasized intricate morphology and compositional harmony.

#### Integration and cross-validation with SEM results.

The visual content analysis serves a critical role in cross-validating the subjective responses observed in the SEM analysis. By triangulating objective expert ratings with participant perception data, we can confirm the validity of the Visual Aesthetic Features (VAF) construct.

Validation of “Symmetry” and Cognitive Mastery: In the SEM results, the path from VAF to Cognitive Mastery was highly significant (β = 0.51). The expert visual analysis provides the explanatory ground for this relationship: the heatmap (Fig 5) reveals that the stimuli received the highest ratings for Symmetry & Balance (M = 4.11). This objective presence of high formal order provides supporting evidence for participants’ ability to cognitively engage with the stimuli despite their novelty. The expert analysis further indicates that the Visual Aesthetic Features (VAF) latent variable adequately captured the structural coherence of the generated images.

Validation of “Novelty” and Emotional Arousal: Similarly, the SEM showed a strong impact of VAF on Emotional Arousal (β = 0.49). Cross-referencing this with the expert coding, the stimuli scored high on Novelty (M = 3.57) and Visual Complexity (M = 3.49). This alignment demonstrates that the arousal reported by participants was not random, but directly triggered by the specific visual attributes (unconventional forms and intricate details) verified by the experts.

Therefore, the visual content analysis functions as a manipulation check, confirming that the AI-generated stimuli possessed the requisite aesthetic properties to validly test the proposed theoretical model [41].

## Discussion

### The role of aesthetic cognition in AI art acceptance

The findings of this study underscore the pivotal role of aesthetic cognition in shaping public acceptance of AIGC-generated urban sculptures. As evidenced by the structural model, visual aesthetic features—including symmetry, complexity, novelty, and symbolic meaning—serve as primary inputs that stimulate both cognitive mastery and emotional arousal, which in turn jointly influence perceived artistic value and ultimately drive artistic acceptance intention.

This sequential pathway aligns closely with Leder et al.’s (2004) five-stage model of aesthetic appreciation, particularly emphasizing the transitional dynamics from perceptual analysis to cognitive mastering. Our results demonstrate that when participants encounter visually novel and compositionally balanced sculpture forms, they are more likely to engage in interpretive efforts to understand the underlying artistic intent. The significant path from visual aesthetic features to cognitive mastery (β = 0.51, p < 0.001) validates the role of perceptual coherence in facilitating semantic elaboration, even in the context of non-human creative authorship.

A particularly important theoretical implication emerges from the role of the cognitive mastery construct. The relatively modest mean score (M = 3.03), which was the lowest among all measured variables, indicates that participants did not achieve a high level of conceptual understanding when evaluating the abstract AIGC-generated forms. Rather than signaling a failure of the construct, this result aligns with the notion that abstract or unfamiliar artworks often elicit ambiguity, perceptual uncertainty, and exploratory meaning-making rather than coherent narrative interpretation. In such contexts, cognitive mastery reflects the attempt to resolve novelty, structural irregularity, or semantic openness, rather than the attainment of a definitive interpretive solution.

Importantly, the SEM results show that this interpretive effort remains a significant positive predictor of perceived artistic value (β = 0.34, p < 0.001). This suggests that, for novel AIGC artworks, aesthetic appreciation may arise not from successful comprehension but from the cognitive engagement stimulated by ambiguity itself. In other words, the viewer's struggle to make sense of unfamiliar AI-generated forms becomes part of the aesthetic experience, consistent with theoretical perspectives that emphasize metacognitive reflection and the rewarding nature of cognitive challenge in contemporary and abstract art. This interpretation acknowledges the limitations of the cognitive mastery construct when applied to highly abstract stimuli while simultaneously demonstrating its relevance as a mechanism of aesthetic valuation.

Moreover, the expert visual analysis confirmed that certain visual features—particularly symmetry and balance (M = 4.11) and meaningfulness (M = 3.55)—played a critical role in anchoring perception and guiding interpretation. These dimensions correspond to the early and middle stages of aesthetic processing, wherein formal fluency supports semantic decoding. In this regard, aesthetic cognition acts as a stabilizing force that enables viewers to negotiate the unfamiliarity of algorithm-generated forms and attribute cultural or symbolic value to them [42].

Theoretically, these findings contribute to an expanded understanding of aesthetic cognition in the age of machine creativity. While classical models were developed in the context of human-made art, this study demonstrates their applicability—and adaptability—to AI-generated content. Importantly, it affirms that cognitive engagement remains essential for the legitimation of artistic value, regardless of the origin of the creative act.

Practically, the results suggest that the design of AIGC-generated public art should prioritize not only surface-level visual appeal but also cognitive affordances that support interpretability, cultural resonance, and symbolic richness [43]. By doing so, designers and policymakers can enhance public engagement and acceptance of AI-generated aesthetics in civic spaces.

In sum, aesthetic cognition functions as the interpretive bridge that transforms perceptual input into evaluative judgment and acceptance. Its role is especially critical in contexts where authorship and authenticity are in question, as is often the case with algorithmically produced art [44]. This study provides empirical evidence that viewers are willing to engage cognitively with AI-generated artworks—provided that the visual stimuli offer sufficient structure, depth, and symbolic cues to invite such engagement.

### Trust, emotional engagement, and artistic value

Beyond cognitive processing, the findings reveal that emotional engagement and trust in AI systems constitute indispensable components in the formation of perceived artistic value and subsequent acceptance of AIGC-generated sculptures. As the structural model indicates, emotional arousal exerts a direct influence on perceived artistic value (β = 0.40, p < 0.001), affirming that affective resonance plays a crucial role in legitimizing algorithmically created art. At the same time, trust in AIGC significantly predicts acceptance (β = 0.22, p < 0.001), functioning as a cognitive-attitudinal moderator that mediates between technological unfamiliarity and public receptivity.

This dual-pathway model highlights an important insight: aesthetic appreciation of AI-generated art is not purely intellectual; it is shaped by visceral affective responses and broader attitudinal beliefs regarding the agency and credibility of artificial creators. The relatively high emotional arousal scores (M = 3.08) suggest that many AIGC-generated sculptures were able to evoke curiosity, wonder, or imaginative stimulation—core emotions known to amplify attention and memorability in aesthetic contexts.

The expert heatmap analysis also substantiates this mechanism. Sculptures scoring high in novelty (M = 3.57) and perceived meaning (M = 3.55) were more likely to elicit emotionally engaging responses, even when their cognitive interpretability remained moderate. These features—particularly unexpected forms, symbolic abstraction, or biomorphic structures—appear to act as aesthetic triggers that stimulate emotional intensity despite the absence of traditional authorship. Such resonance indicates that the human emotional system is responsive not only to the origin of the artwork but also to its form-driven capacity to surprise, inspire, or provoke reflection [45].

Trust, on the other hand, functions as an attitudinal gateway for reconciling the novelty of AI-produced artworks with existing cultural expectations around creativity and authenticity. While prior literature often highlights skepticism toward AI in cultural domains (Oprea et al., 2024) [46], our findings suggest a more nuanced view: audiences are willing to extend aesthetic legitimacy to AI-generated art when they perceive the system as capable of producing original, ethical, and meaningful content. Trust thus serves as a cognitive scaffolding that enables audiences to bridge the ontological gap between human and non-human creators.

Importantly, the coexistence of emotion-driven and trust-based acceptance mechanisms reflects the hybrid nature of contemporary aesthetic experience in the era of generative AI. While traditional art perception models emphasized either cognitive elaboration or emotional response, our integrated framework shows that acceptance of AI art requires a confluence of aesthetic stimulation, emotional resonance, and techno-cultural trust. The stronger these components, the more likely viewers are to perceive AI-generated sculptures as credible artistic expressions worthy of public display [47].

From a design perspective, this suggests that successful AIGC artworks should strategically balance visual novelty (to activate emotion), symbolic or cultural familiarity (to enhance meaning), and transparent authorship narratives (to foster trust). For instance, integrating brief descriptions of the AI's generative logic or its collaboration with human curators may help mitigate skepticism and deepen audience engagement.

### Theoretical implications: Extending aesthetic models

The results of this study offer several theoretical contributions to the evolving field of empirical aesthetics and human–AI interaction, particularly by extending classical aesthetic processing models into the domain of artificial intelligence–generated public art. Traditional models of aesthetic experience, such as Leder et al.’s (2004) five-stage framework, have been primarily validated in contexts involving human-created art forms—paintings, photographs, or architecture—where authorship, intentionality, and cultural context are often implicitly understood. By applying and empirically validating this model within the realm of AIGC-generated sculptures, our findings demonstrate that these foundational theories remain applicable, yet require important modifications when situated in post-human creative environments.

First, our integrated framework confirms the continued salience of perceptual, cognitive, and affective mechanisms in shaping aesthetic judgments. However, it also reveals that additional variables such as “trust in artificial authorship” must be incorporated to fully capture evaluative processes in AI-generated art contexts. This suggests that aesthetic experience in the digital age is no longer reducible to formal features or viewer expertise alone; rather, it is co-constructed through the interplay of visual stimuli, cognitive effort, emotional response, and attitudinal dispositions toward non-human agency.

Second, the strong predictive role of trust and familiarity in artistic acceptance highlights the need to expand aesthetic models to include sociotechnical constructs. As generative systems increasingly take on roles traditionally reserved for human artists, audiences must not only evaluate what they see, but also reconcile how and by whom it was created. This calls for a shift from a purely object-centric understanding of aesthetic value toward a process-aware model, where the viewer's interpretation is shaped by both the artifact's visual qualities and its generative ontology [48].

Third, the demonstrated interaction between cognitive mastery and emotional arousal as parallel mediators of artistic value offers support for dual-process theories of aesthetic judgment, where both rational interpretation and affective resonance contribute to meaning construction. This aligns with emerging neuroaesthetic frameworks that view aesthetic experience as a dynamic integration of top-down cognitive appraisal and bottom-up sensory-affective processing [49]. Our findings enrich this discourse by showing that such dual mechanisms persist even when the artwork is non-anthropogenic in origin.

Fourth, this study provides empirical evidence for the legitimation of machine-generated artifacts as objects of aesthetic experience. The fact that participants were able to form coherent judgments of beauty, meaning, and artistic legitimacy for AI-generated sculptures supports the notion that aesthetic cognition is medium-agnostic—that is, it can operate effectively even when traditional assumptions about artistic creation are disrupted.

Importantly, our findings also resonate with recent theoretical discussions concerning unresolved questions in AI aesthetics, particularly those articulated in recent scholarship published in Frontiers in Psychology [50]. That work called for empirical clarification regarding whether established models of aesthetic appreciation remain valid in contexts where creative agency is attributed to artificial systems rather than human artists. The present study contributes to this discourse by demonstrating that perceptual, cognitive, and affective mechanisms continue to function in structurally coherent ways within AI-generated art evaluation. At the same time, our results indicate that trust in artificial authorship operates as an additional evaluative dimension that meaningfully shapes acceptance intentions.

Rather than suggesting a replacement of classical aesthetic frameworks, our findings support a model of theoretical extension, wherein traditional cognitive-emotional appraisal mechanisms remain foundational but are complemented by trust-based considerations specific to AI-mediated creativity. In this sense, the current study provides empirical grounding for ongoing theoretical efforts to refine models of aesthetic judgment in the era of generative artificial intelligence.

### Practical implications for urban art design and planning

The empirical insights derived from this study hold significant implications for the design, curation, and implementation of AIGC-generated art in urban public spaces. As cities increasingly explore the integration of generative technologies into their cultural infrastructures, understanding how the public perceives, evaluates, and accepts AI-generated sculptures becomes vital for effective and sustainable cultural planning.

First, the positive influence of visual aesthetic features—particularly symmetry, complexity, novelty, and perceived meaning—on cognitive and emotional engagement suggests that urban planners and designers should prioritize these qualities when selecting or generating AI-based public artworks. Rather than focusing solely on technical novelty or formal abstraction, public art initiatives should aim to balance visual coherence with symbolic richness, ensuring that artworks are both emotionally resonant and cognitively interpretable to diverse audiences.

Second, the study highlights the importance of audience trust in AI-generated art. Public art policies should therefore include educational and communicative strategies that promote transparency regarding the generative process. For example, including explanatory signage, QR codes linking to the generative algorithm, or artist–AI collaboration narratives can help demystify the creation process and foster greater acceptance. These interventions not only mitigate skepticism but also encourage viewers to reflect on the evolving nature of creativity in the age of artificial intelligence [51].

Third, the significant role of emotional arousal in enhancing perceived artistic value and acceptance intention indicates that AIGC public artworks should be designed to evoke curiosity, surprise, or wonder—emotions known to enhance memorability and participatory engagement in public space. This calls for experience-centered design strategies, such as interactive installations, immersive lighting effects, or site-responsive adaptations, which can elevate emotional resonance and situational relevance.

Fourth, the study shows that even viewers unfamiliar with AIGC technologies are capable of engaging meaningfully with AI-generated sculptures when visual stimuli are well-crafted. This suggests that inclusivity in design does not require simplifying content, but rather curating works that offer multiple entry points for interpretation—whether through form, theme, or cultural association [52].

Finally, the validated conceptual framework presented here can serve as a diagnostic tool for future public art evaluation. Urban authorities, cultural institutions, and design teams can adapt this model to assess public responses to AI-generated installations, enabling data-informed decision-making regarding placement, style, and audience alignment. Such an approach supports more participatory, transparent, and socially attuned practices in the deployment of digital creativity within the urban fabric.

In summary, the successful integration of AIGC-generated sculptures into public space hinges not only on their technical sophistication but on their ability to evoke meaningful experiences, foster emotional resonance, and establish trust. This study provides actionable insights for urban designers, artists, and policymakers to guide the ethical, aesthetic, and participatory deployment of algorithmic art in future cityscapes.

### Limitations and future research directions

While this study provides a validated model of the psychological mechanisms underlying AIGC art acceptance, several limitations must be acknowledged, each pointing to important directions for future research.

First, a primary methodological limitation lies in our measurement of the exogenous construct Visual Aesthetic Features (VAF), which relies on subjective, self-reported perceptions. Although such perceptual measures are appropriate for capturing phenomenological aesthetic experience and align with the stages of Leder et al.’s information-processing model, they do not objectively quantify the specific visual attributes of the stimuli that may underlie these responses. Future studies would benefit from integrating computational aesthetics techniques capable of extracting quantifiable image-level descriptors. For example, measures such as visual complexity and entropy [53], chromatic contrast and heterogeneity [54], and compositional proportion or balance [55] can be algorithmically derived from generative outputs. Correlating these objective, low-level visual metrics with the higher-level psychological constructs modeled in this study (VAF, cognitive mastery, emotional arousal) would enable a more precise characterization of the stimulus-to-cognition pathway. Moreover, such integration would make the framework applicable to generative design workflows, supporting the systematic creation of AI artworks optimized for specific perceptual or affective outcomes.

Second, as foregrounded in the Introduction, the ecological validity of the study is inherently limited by its reliance on two-dimensional digital renderings rather than physical sculptures situated in real urban environments. This methodological constraint introduces a substantial gap between viewing digital representations and experiencing public art in situ, where multisensory factors—including materiality, scale, lighting conditions, spatial immersion, and socially situated interactions—play decisive roles in aesthetic evaluation. Consequently, the findings should be interpreted as preliminary insights into public responses to conceptual AIGC artworks and should not be directly generalized to on-site public art installations or urban design decision-making. Future research should adopt immersive methodologies such as VR/AR-based spatial simulation, full-scale prototyping, or field-based studies once physical AIGC sculptures are more widely realized.

Third, the study adopted a cross-sectional design, collecting data at a single point in time. While Structural Equation Modeling (SEM) provides robust estimates of path coefficients, it relies on theoretical assumptions to infer directionality and cannot strictly prove causal relationships between latent variables. For instance, while we posit that visual features influence emotional arousal, it is theoretically possible that heightened emotional states could also bias visual perception. This limitation is inherent to cross-sectional survey methodologies, as noted in similar large-scale investigations of psychological responses in digital or educational contexts [56]. Future research should therefore consider employing longitudinal designs or experimental protocols (e.g., manipulating specific visual cues in a controlled lab setting) to definitively establish causality and track how aesthetic acceptance evolves over time.

### Societal and ethical implications

In addition to the psychological mechanisms and acceptance pathways examined in this study, the broader societal and ethical implications of integrating AIGC technologies into public art must also be acknowledged. While generative systems offer opportunities for expanding creative possibilities, their adoption carries potential risks that warrant careful consideration.

One concern relates to the shifting role of human artists within increasingly algorithm-mediated creative environments. The widespread use of AIGC tools may contribute to the deprofessionalization of artistic labor, potentially diminishing the cultural and economic value traditionally associated with human craftsmanship and intentionality. This raises important questions about how artistic authorship, originality, and creative agency should be understood and protected in an era when machine-generated outputs can closely approximate human artistic production.

Moreover, reliance on generative models introduces the risk of algorithmic aesthetic homogenization. Because these systems are trained on large-scale datasets that reflect existing visual patterns and biases, the resulting artworks may inadvertently reproduce dominant stylistic conventions while marginalizing diverse cultural expressions. Such homogenization could limit the plurality and contextual specificity that are central to public art's role in representing local identity and fostering cultural dialogue.

These considerations underscore the need for balanced and responsible deployment of AIGC technologies in public spaces. Audience acceptance, while essential, should not overshadow the ethical imperative to preserve human creativity, ensure cultural inclusivity, and develop regulatory frameworks that safeguard the integrity of artistic practice.

## Conclusion

This study investigated the aesthetic cognition and artistic acceptance of AIGC-generated urban sculptures by integrating a structural equation modeling (SEM) approach with expert visual content analysis. In addressing the central question of how viewers cognitively and emotionally engage with algorithmically created public artworks, we developed and validated a conceptual framework that encompasses perceptual features, cognitive mastery, emotional arousal, perceived artistic value, trust in AIGC, and artistic acceptance intention.

The empirical findings reveal several key insights. First, visual aesthetic features—notably symmetry, complexity, novelty, and symbolic meaning—serve as foundational inputs that significantly influence both cognitive interpretation and emotional engagement. These perceptual and affective pathways jointly shape the viewer's perception of artistic value, which in turn emerges as the strongest predictor of public acceptance of AI-generated sculptures. Second, trust in AIGC plays a meaningful and independent role in shaping acceptance, suggesting that the perceived legitimacy of non-human creative agents is crucial in the context of public art. Third, despite the relative novelty of AIGC outputs, the audience demonstrated a moderate-to-positive inclination to accept such works, especially when aesthetic coherence and symbolic depth were present.

Theoretically, this research extends established aesthetic cognition models—particularly Leder et al.’s five-stage framework—into the realm of algorithmically produced artworks. By introducing trust as an additional cognitive-attitudinal construct, the study adapts classical theories to contemporary contexts where creativity is no longer exclusively human. The findings support a triadic model of aesthetic evaluation that integrates perception, affect, and trust, offering a new lens through which to study aesthetic experience in technologically mediated environments.

Methodologically, the study contributes an innovative mixed-methods approach that combines AI-based image generation (Midjourney v6), expert visual coding, and psychometric modeling, thereby providing a replicable template for future research on AI aesthetics, digital design evaluation, and public perception studies. The validated model and visual stimuli library can be used for cross-cultural, longitudinal, or comparative investigations across different artistic media or urban contexts.

Practically, the findings offer actionable guidance for urban designers, cultural planners, and policy makers seeking to integrate AIGC into public art initiatives. Emphasizing emotionally engaging, cognitively interpretable, and visually coherent designs—while fostering transparency and trust in AI authorship—can enhance the cultural legitimacy and public resonance of algorithmic artworks in civic spaces.

Future research may expand on this study by exploring longitudinal changes in public attitudes toward AIGC art, examining cross-cultural differences in trust and aesthetic preference, or investigating how hybrid human–AI co-creation models are perceived compared to fully autonomous generative systems. Additionally, neuroaesthetic or biometric approaches (e.g., eye-tracking, EEG) could be employed to further uncover the unconscious processes underlying AI art perception.
